# Editorial on the Research Topic the 2nd Edition of Mountain Sports Activities: Injuries and Prevention

**DOI:** 10.3390/ijerph19159510

**Published:** 2022-08-02

**Authors:** Martin Burtscher, Urs Hefti, Gerhard Ruedl, Jacqueline Pichler Hefti

**Affiliations:** 1 Department of Sport Science, University of Innsbruck, 6020 Innsbruck, Austria; gerhard.ruedl@uibk.ac.at; 2 Austrian Society for Alpine and High-Altitude Medicine, 6020 Innsbruck, Austria; 3 Swiss Sportclinic, 3014 Bern, Switzerland; urs.hefti@swiss-sportclinic.ch (U.H.); jacquelinepichler@gmx.net (J.P.H.); 4 Medical Commission, International Climbing and Mountaineering Federation (UIAA), 3014 Bern, Switzerland

## 1. Introduction

Mountain sports are continuously gaining popularity, currently fueled by the post-pandemic period expanding travel opportunities and the desire to escape the increasingly hot environmental conditions of urban areas—ambient temperature decreases by about 6.5 °C per 1000 m gain in altitude. Although mountain sports activities are contributing to the well-accepted beneficial health effects of physical activity [1], associated risks from injury and cardiovascular events, and therefore the net health benefits, vary largely between different types of mountain sports [2,3]. As true for almost every type of sport [4], some health risk may arise related to a particular sport, individual behavioral aspects and health status. For example, the annual death rate may amount to less than one in one million downhill skiers (performing their sport on well-prepared and secured slopes) [3], but may increase more than 100-fold in rock and ice climbers in remote areas or high-altitude expeditioners [2]. Whereas young people are mostly affected by traumatic events, e.g., falls, older individuals are at of both traumatic and non-traumatic (primarily cardiovascular) events [2,3]. The risks associated with easy-to-perform activities, e.g., mountain hiking, are generally underestimated, representing the major proportion of accidents occurring in the mountains during the summer season [5,6]. Knowledge on risk factors which are associated with injuries or (non-traumatic) emergencies is of utmost importance for the successful implementation of preventive interventions. Furthermore, validation and continuous revalidation of such measures are necessary.

## 2. Some Risks Are Modifiable

Generally, risk factors can be divided into intrinsic (related to the individual) and extrinsic (environmental) factors [7]. Examples of intrinsic factors are age, sex, body mass, physical fitness, behavioral aspects (e.g., risky behavior), and extrinsic factors include weather and terrain conditions (e.g., falling rocks and ice), high altitude exposure, the type and level of preparation of hiking paths and skiing slopes. More importantly however, is the consideration of whether risk factors can be modified or not. For instance, the individual fitness, specific alpine skills, and knowledge about risks are largely modifiable and appropriate adaptations can contribute to the prevention of both traumatic and non-traumatic events [8,9].

## 3. Examples for Research Ideas

The development of effective strategies for the prevention of accidents/emergencies in mountain sports requires, beside updated knowledge derived from observational (epidemiological) studies, the inclusion of up-to-date findings from basic research. For example, information on cardiovascular and musculo-skeletal strain during hiking, climbing, skiing, cycling, etc., under different ambient conditions (e.g., altitude, cold, heat) is extremely helpful to advise healthy and in particular diseased individuals on the selection of and how to prepare for mountain sports activities [10,11,12,13,14]. Furthermore, knowledge on pathophysiological mechanisms triggering high-altitude illnesses (e.g., acute mountain sickness, high-altitude cerebral and pulmonary edema) is a prerequisite for the prevention and appropriate treatment of those illnesses as well [15,16,17].

Research will also have to counter new dangers arising from climate change. For example, many classical routes will no longer be climbable to the same extent as they are nowadays; the thawing process of dead ice and the receding glaciers will create completely new hazards. There will also be a shift in the climbing season, and known weather phenomena (e.g., the monsoon in the Himalayas) will become less predictable and possibly more severe in their impact—which are factors that need to be considered in our prevention efforts.

Finally, the development of constantly improving protective equipment and the implication of optimized rescue possibilities are further examples of potential research areas contributing to the improvement of the net health benefits from mountain sports activities [18,19,20].

## 4. Key Focus of Contributions

Thus, this Special Issue “The 2nd edition of Mountain Sports Activities: Injuries and Prevention” will not only include observational findings, but will particularly focus on interventional studies tackling those novel challenges due to climate change and the greater participation of elderly and diseased individuals in mountain sports. In addition, findings from a broad range of basic research may provide extremely valuable support for the implementation of preventive and treatment strategies regarding both traumatic and non-traumatic events potentially occurring during (all kinds of) mountain sports activities. Of course, comprehensive reviews and exceptional case reports may add valuable contributions.
